# Prompt Identification of Sepsis on Hospital Floors: Are Healthcare Professionals Ready for the Implementation of the Hour-1 Bundle?

**DOI:** 10.3390/tropicalmed7100291

**Published:** 2022-10-10

**Authors:** Sadia Shakeel, Wajiha Iffat, Shagufta Nesar, Sidra Shayan, Aatka Ali, Márió Gajdács, Shazia Jamshed

**Affiliations:** 1 Department of Pharmacy Practice, Faculty of Pharmaceutical Sciences, Dow College of Pharmacy, Dow University of Health Sciences, Karachi 74200, Pakistan; 2 Department of Pharmaceutics, Faculty of Pharmaceutical Sciences, Dow College of Pharmacy, Dow University of Health Sciences, Karachi 74200, Pakistan; 3 Jinnah College of Pharmacy, Sohail University, Karachi 74000, Pakistan; 4 Department of Pharmaceutics, Faculty of Pharmacy, Hamdard University, Madinat al-Hikmah, Hakim Mohammad Said Road, Karachi 74600, Pakistan; 5 Department of Oral Biology and Experimental Dental Research, Faculty of Dentistry, University of Szeged, 6720 Szeged, Hungary; 6 Clinical Pharmacy and Practice, Faculty of Pharmacy, Universiti Sultan Zainal Abidin, Kuala Terengganu 21300, Malaysia

**Keywords:** sepsis, Surviving Sepsis Campaign, bundles, healthcare professionals, healthcare systems, physicians, knowledge, emergency medicine, developing country, Pakistan

## Abstract

Early intervention in sepsis management with recognized therapeutic targets may be effective in lowering sepsis-related morbidity and mortality, although this necessitates timely identification of sepsis by healthcare professionals. The present study aimed to assess knowledge levels, attitudes, and agreement among physicians regarding the Surviving Sepsis Campaign (SSC) guidelines (more specifically, the Hour-1 bundle). A quantitative, descriptive, cross-sectional study was conducted among physicians working in different clinical settings in Karachi, Pakistan, using a self-administered questionnaire. The mean cumulative knowledge score of the respondents towards SSC was 6.8 ± 2.1 (out of 10), where a total of *n* = 127 respondents (51.62%) had a strong understanding of the SSC guidelines, compared to *n* = 78 (31.7%) and *n* = 41 (16.7%) respondents with fair and inadequate knowledge, respectively. The majorly known bundle elements by the respondents were to administer broad-spectrum antibiotics (89.8%, *n* = 221), the need for taking blood cultures before administering antibiotics (87.8%, *n* = 216), and measurement of blood lactate levels (75.6%, *n* = 186). Experienced physicians were more likely to use norepinephrine as the first-choice vasopressor (*p* < 0.001). Female respondents were more likely to consider the duration of antibiotic therapy to be determined according to the site of infection, the microbiological etiology, the patient’s response to treatment, and the likelihood of achieving adequate source control (*p* = 0.001). The current study concluded that respondents had an optimistic approach and frequently practice in accordance with the SSC guidelines, while some respondents were not up to date with the most recent guidelines. There is a need for further interventions and continuous medical education to encourage physicians towards appropriate use of the recommended guiding principles for improving treatment outcomes in sepsis patients.

## 1. Introduction

Sepsis is one of the most frequently reported health concerns in intensive care units (ICUs), corresponding to considerably high morbidity and mortality rates all over the globe [1]. Sepsis is a life-threatening emergency, especially in low and middle-income countries (LMICs), where it is a significant contributor to neonatal and maternal mortality, in addition to disproportionally affecting elderly, immunocompromised, and severely ill patients [2]. Hundreds of millions of patients worldwide experience sepsis annually, as a result of infections contracted in medical facilities (i.e., healthcare-associated infections, HAIs), which are one of the most common adverse outcomes during the provision of care [3]. Sepsis negatively affects the patients’ physiology and psychology, and may result in multiple organ dysfunction (MODS) and failure (MOF). Untreated sepsis may lead to septic shock, respiratory distress syndrome, acute renal failure, disseminated intravascular coagulation (DIC), cardiac arrhythmia (e.g., atrial fibrillation), and MOF [4]. Moreover, surviving sepsis has been linked to post-traumatic stress disorder, depression, and anxiety. Sepsis patients usually experience prolonged ICU stays, and they face an extended, complex path to recovery. Besides the physical recovery challenges, patients and their families are often not sure how to accomplish their goal of care [1]. The decision-making practice for the timely detection and control of sepsis is critical for frontline healthcare professionals. It has been described that improvements in professional behaviors, attitudes, and knowledge about the prompt diagnosis and treatment are associated with improved patient outcomes [3]. Previous studies have demonstrated that misinterpretation and misunderstanding of the clinical manifestations of sepsis, as well as insufficient training towards management protocol among healthcare professionals, might negatively affect the treatment outcomes of affected patients [2,5,6].

The Surviving Sepsis Campaign (SSC) is a global platform that developed guiding principles to advance the treatment for reduced morbidity and mortality rates associated with sepsis [7]. Numerous studies have shown that the scientific application of these bundle features improves sepsis care and have led to decreased mortality rates [8,9]. In response to the accomplishment of “Surviving Sepsis Campaign: International Guidelines for Management of Sepsis and Septic Shock: 2016”, a reviewed “Hour-1 bundle” was established [10]. Recent international adult sepsis guidelines—released in October 2021—better emphasize on effective care of sepsis patients once they get discharged from the ICU [8]. The updated guidelines represent better geographical and gender diversity than earlier versions, and precisely address the difficulties of treating patients facing the long-standing effects of sepsis [8]. To overcome these issues, the guiding principles recommend relating patients and their caregivers to the targeted goals-of-care, which include continuing follow-up with physicians to support and accomplish lasting consequences and evaluation of physical, mental, and cognitive problems upon discharge [7]. After the recent updates in sepsis medical care, little is known regarding physicians’ knowledge and agreement with the implementation of the SSC guidelines into their everyday practice [7]. Therefore, to determine prospects of sepsis-related clinical research and practice, it is imperative to evaluate healthcare professionals’ knowledge and understanding of sepsis among critical care patients [11]. With this in mind, the current research was conducted among physicians having an experience of working in ICUs in Karachi, Pakistan, to assess their levels of knowledge, attitude, and agreement regarding the the most recent SSC guidelines.

## 2. Materials and Methods

### 2.1. Study Design, Sample Size Determination

A quantitative, descriptive, cross-sectional study was conducted from May to November 2021 in Karachi, Pakistan. The target population involved physicians, particularly those having an experience of working with ICU patients in four public and five private healthcare facilities in Karachi. The respondents were chosen using the convenience and snowball sampling methods, based on their proximity and ease of availability for inclusion. The respondents were considered eligible to participate in the study provided they were registered with the professional body for accreditation and filled a written consent form for their voluntary contribution to the study. The minimum required sample size for the study was determined by the Raosoft sample size calculator (Raosoft Inc., Seattle, DC, USA), described previously [12]; overall, the sample size was determined as *n* = 377.

### 2.2. Instrument Development

A questionnaire was developed after reviewing the relevant literature on the topic [3,5,10,13] with the opinions of experts, including two infectious disease specialists and five senior ICU physicians. To establish content validity of the instrument, the questionnaire was pre-tested in a small subset of physicians (*n* = 30) to evaluate the transparency and clarity of question items (face validity). The Hoyt-reliability scale was used to assess reliability quantitatively; the reliability score was found to be 0.781. Based on expert opinions and the comments of the physicians in the pre-test phase, the final questionnaire was developed.

The final questionnaires were then circulated among the relevant physicians through direct correspondence or e-mail (for those whose e-mail addresses were available) after describing to them the rationale and aims for conducting the research. After providing the respondents with information on the study’s goals, advantages, and risks, they were provided with a questionnaire, which was self-administered. The respondents filled out the questionnaires in an anonymous manner. According to the convenience of the respondents, the questionnaires and the consent form were gathered afterward. The questionnaire consisted of the following parts: *(i)* a demographic portion (with six questions overall), *(ii)* 30 questions addressing their knowledge, attitude, and agreement with regard to the “Surviving Sepsis Campaign: International Guidelines for Management of Sepsis and Septic Shock.” [8]. The comprehension of the respondents was scored “1” for each correct answer and “0” for each incorrect, “Don’t know”, or “Unsure” answer. As there were ten questions used overall to assess respondents’ knowledge, to determine the cumulative knowledge score (SSC), the maximum SSC was 10. Each respondent’s overall percent knowledge (score obtained/SSC × 100) was calculated, and their understanding of the SSC was rated as good (score ≥ 70), fair (score 50.1–69.9), or low (score ≤ 50). The third *(iii)* part of the questionnaire consisted of 15 questions that used a 5-point Likert scale (ranging from “Strongly disagree” to “Strongly agree”) to examine the respondents’ attitude toward the SSC Bundle in their clinical practice. Finally, (*iv*) additional questions were included about the respondents’ perceived causes behind the increasing rate of sepsis, their recommendations for setting goals of care for sepsis patients, and their perceived barriers to using the SSC Bundle in practice.

### 2.3. Statistical Analyses

All continuous variables were expressed as means and standard deviations (SD), whereas categorical variables were expressed as frequencies (*n*) and percentages (%). The χ^2^-square test and Student’s t-test were used to associate the demographic characteristics among respondents who had knowledge of SSC and those who were not knowledgeable about SSC. Statistical analyses were performed using SPSS Statistics version 24.0 (IBM Inc., Chicago, IL, USA). During analyses, *p* values < 0.05 were considered statistically significant.

### 2.4. Ethical Considerations

Ethical approval for this study was obtained from the Institutional Review Board of Liaquat College of Medicine and Dentistry, Darul Sehat Hospital, Karachi, Pakistan (Reference No. DSH/IRB/2021/0027).

## 3. Results

### 3.1. Demographic Characteristics

Out of the 384 survey forms that were distributed among the physicians, *n* = 246 physicians consented to participate in our study and completed the survey, corresponding to a response rate of 64.0%. Table 1 summarizes the demographic characteristics of the respondents. The mean age of respondents was 42.7 ± 3.5 years, with the number of male respondents being in a majority (56.9%, *n* = 140). Over two-thirds (68.2%, *n* = 168) of the participants were rendering their services in private hospitals/clinics. Ninety-three (37.8%) respondents stated that they observe one to five sepsis patients/ month, whereas *n* = 58 (23.5%) and *n* = 95 (38.6%) see five to ten and more than ten sepsis patients, respectively.

### 3.2. Physicians’ Knowledge Regarding the Surviving Sepsis Campaign Bundle Elements

The mean cumulative knowledge score of physicians’ SSC was 6.8 ± 2.1. A total of *n* = 127 respondents (51.62%) reported having a strong understanding of the SSC guidelines, compared to *n* = 78 (31.7%) and *n* = 41 (16.66%) who had fair or inadequate knowledge, respectively. The majority of the respondents (76.0%, *n* = 187) were familiar with the definitions of sepsis and septic shock as per the SSC Bundle. The majorly known bundle elements by the respondents were to administer broad-spectrum antibiotics (89.8%, *n* = 221), the need for blood culture before the antibiotic administration (87.8%, *n* = 216), and measurement of blood lactate levels (75.6%, *n* = 186) (Table 2).

### 3.3. Physicians’ Attitude towards the Surviving Sepsis Campaign Bundle

The summary of responses for the attitude questions are presented in Table 3. Around 83% of the respondents believed that sepsis is a medical emergency requiring immediate attention, and they have considered it an important reason for mortality as compared to other disease conditions. More than 80% of physicians agreed that intravenous (IV) administration of antibiotics should be initiated at the earliest possible time (or within 60 min) after the diagnosis of the disease. Senior physicians with 11–15 years of experience acknowledged that pharmacokinetic/pharmacodynamic standards and explicit medication properties improve dosing techniques of antibiotics (*p* = 0.003). In addition, they highlighted norepinephrine as the first-choice vasopressor (*p* < 0.001). In contrast, dopamine was considered as an alternate agent to norepinephrine merely in very carefully chosen patients (*p* = 0.003). Female respondents were more likely to consider the duration of antibiotic therapy to be determined according to the site of infection, the microbiological etiology, the patient’s response to treatment, and the likelihood of achieving source control (*p* = 0.001).

### 3.4. Physicians’ Perceived Reason behind the Increase in Sepsis Incidence

**Figure 1:**
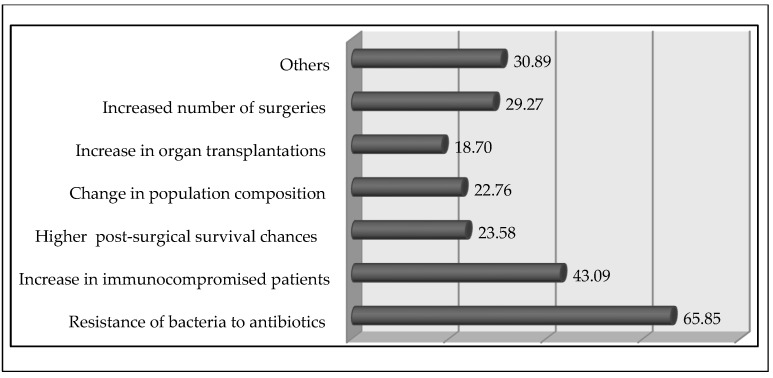
Physicians’ perceived reasons for the increased incidence rate of sepsis (%).

More than half (51.2%, *n* = 126) of the physicians thought that the incidence had been increasing steadily, whereas (17.1%, *n* = 42) believed that it had been increasing dramatically (*p* = 0.028) in the last five years of their clinical practice. The majority of the respondents (65.8%, *n* = 162) considered the increased resistance of bacteria to antibiotics as the major reason for the increased incidence rate (Figure 1). Most (85.3%, *n* = 210) respondents emphasized that a drop in mortality rate may be observed after implementing the SSC protocol. On inquiring about the way to deal with sepsis, *n* = 159 (64.6%) respondents thought that the most significant treatment approach is fluid replacement, whereas *n* = 61 (24.7%) and *n* = 26 (10.5%) respondents stated antimicrobial treatment and inotropic support are the most significant parts of patient management, respectively.

### 3.5. Physicians’ Recommendations for Setting Goals for the Care of Sepsis Patients

**Figure 2:**
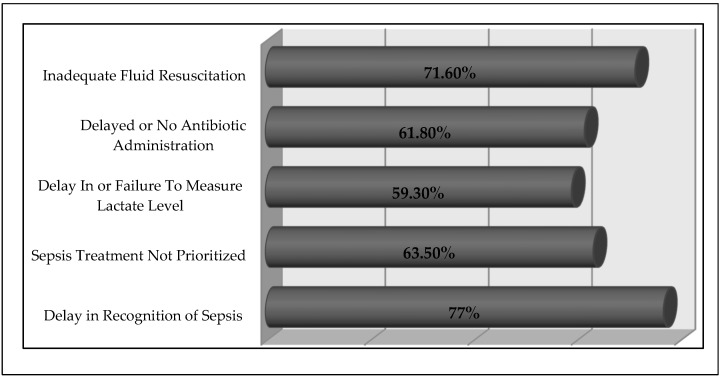
Physicians’ perceived barriers for incorporating SSC bundles in their practice.

On inquiring about the physicians’ recommendations for setting goals of care for sepsis patients, it was observed that 15% (*n* = 37) of the physicians recommended that patients and their families should be communicated about the objectives of the care plan. A total of 23.9% of respondents suggested that the objectives of care should be integrated into treatment and patient care plan designs, using palliative care ethics wherever applicable. Figure 2 depicts the physicians’ perceived barriers to treat sepsis patients. The majority of respondents (77.2%, *n* = 190) considered delay in the identification of sepsis patients as the primary barrier to their adequate care.

## 4. Discussion

The current study had a response rate of 64.0%. The lower response rate might be because healthcare professionals—particularly physicians treating ICU patients—have time constraints due to demanding work schedules in responding to research/health surveys. The present study aimed to reveal physicians’ knowledge of SSC guidelines, and to identify potential knowledge gaps in sepsis awareness and management. To the best of our knowledge, the present study is the first of its kind from Pakistan evaluating the knowledge of physicians on sepsis using indicators of diagnosis and management mentioned in SSC guidelines. Sepsis is the cause of 60–80 percent of fatalities in underdeveloped nations, such as Pakistan, being a significant burden of disease [14]. The highly disproportionate morbidity and mortality from sepsis in this country may be due to low living standards, poor hygiene, malnutrition, restricted access to healthcare facilities, and structurally weak health systems. It is essential to establish a stronger understanding about how physicians manage sepsis patients and how competent they are in the diagnosis and treatment of sepsis [11]. In the present study, the majority of the respondents were familiar with the definitions of sepsis and septic shock as per the SSC Bundles. The study of Suntornlohanakul et al. revealed that 55.9%, 66.9%, and 94.1% of respondents were able to respond to inquiries about the definitions of severe sepsis, sepsis with severe hypoperfusion, and septic shock, respectively. However, only 32.4% could distinguish between different levels of sepsis severity [15]. According to a Nepalese study, almost 46% of participants who had experience working in ICUs had sufficient understanding about sepsis [16]. In contrast to past research that found healthcare workers to lack appropriate knowledge on sepsis and its management practices [11], our study had a larger proportion of individuals who provided accurate answers. The disparities between the studies could be—at least partly—the result of variations in the corresponding questionnaires used. It necessitates the requirement for practical measures to further expand the degree of awareness of physicians through continuous medical education (CME). For example, a study conducted by Ahmed et al. depicted that 37.9% of the participants were having adequate knowledge [14]; specialty and work experience all substantially predicted knowledge levels regarding sepsis (*p* = 0.0001), with medical residents who were more recently trained and those with more critical care experience displaying greater understanding [14]. Another major outcome of the present study was that the respondents considered sepsis an emergency condition that needs immediate treatment, and they understood the urgency in the resuscitation of critically ill sepsis patients. Husabo et al. reported that among the majority of sepsis patients, critical steps for diagnosing sepsis and organ failure in the emergency room were delayed or missed [17]. On evaluating respondents’ familiarity and adherence to the SSC criteria across different hospital levels, we observed that respondents from private hospitals had higher levels of familiarity (*p* = 0.001). These results demonstrated that the acceptance of the guidelines varied among hospital levels and healthcare institution type. One possible explanation could be that private hospitals have more opportunities to treat critically ill sepsis patients, which encourages them to follow the most recent recommendations. Additionally, private hospitals have better, more advanced medical facilities, which assures that professionals follow the rules and guidelines when conducting therapeutic interventions. Thus, a healthy outlook on practice and learning, as well as a diversity of medical resources, have a significant impact on physicians’ knowledge and skills.

Our respondents showed a positive attitude towards the SSC, and more than three-fourth of respondents emphasized that a reduction in mortality rate can be observed after implementing the SSC protocol. A similar outcome was reported by Faiza et al., in which more than two thirds of the respondents strongly agreed that sepsis continues to be one of the unmet needs in critical care [14]. This is in line with the results of a study from China, which showed a significant drop in mortality after the implementation of the protocol in clinical practice [11]. In the current study, the majority of physicians reported that the incidence of sepsis was increasing and stated that chances of getting sepsis increased linearly with the increase in age, as this could be attributed to increased bacterial resistance to antibiotics. Other studies also reported parallel findings and stated a similar reason for an increased incidence rate of sepsis [18]. More experienced physicians believe that sepsis treatment is one of the most urgent necessities in critical care today. Furthermore, they deemed that the presentation of symptoms in the elderly may be more severe and dissimilar from those in younger individuals. A Malaysian study revealed that younger responders knew more about sepsis than more experienced physicians [19]. This may be owing to differences in the medical curriculum, and the fact that younger responders were mostly residents who may have studied sepsis more recently and had more frequent experiences with septic patients as a result of their extended training hours.

Early detection of sepsis as well as better care of sepsis sufferers in the emergency room are the main factors for the declining mortality rate [8]. The respondents of the current research expressed an identical approach and emphasized that healthcare facilities should follow an improved treatment program to be executed in patients with sepsis, including early screening for critically ill patients. Other investigations have demonstrated that even while standards and guidelines are in place, their adherence is more of a worry and requires routine auditing [11]. Likewise, it is supported by the fact that developing countries may have a scarcity of resources in healthcare to objectify components of the SSC Bundle [20]. For instance, mechanical ventilation and the measurement of blood lactate to characterize sepsis are not easily executable in underdeveloped regions of the world.

The majority of the respondents considered that the increased resistance of bacteria to antibiotics could potentially increase the incidence rate of sepsis. Antimicrobial resistance (AMR) is a significant determinant of clinical treatment resistance and the quick onset of sepsis and septic shock [21]. Treatments that are partial, delayed, or ineffective add to the threat posed by AMR and raise the risk of sepsis in patients. The prevalence of AMR organisms has significant effects on sepsis therapy, especially in countries having inadequate clinical microbiology setups [22]. Sepsis patients with resistant pathogens have been observed to have a higher risk of complications and mortality. Methicillin-resistant *S.* *aureus* (MRSA) is thought to be 50% more likely to cause death in individuals than methicillin-susceptible *S.* *aureus* [23]. The severity of the disease and a proper diagnosis should be considered when determining which patients require rapid and aggressive therapy. Antibiotic therapy should be chosen carefully based on laboratory, radiographic, and microbiological data if a patient has a low probability of infection and is not in shock. It is still up for debate how the guidelines should be operationalized for the timing of antibiotics for patients with sepsis [21]. The guidelines have been criticized for not having a strong enough empirical foundation, and for relying on treatment regimens that mandate the early administration of broad-spectrum antibiotics to all sepsis patients and the beginning of antibiotics within an hour of triage [24]. The Infectious Disease Society of America (IDSA) has commented on the sepsis guidelines and suggested giving patients with serious illnesses antibiotics as soon as possible [25]. However, they caution that strict guidelines with set deadlines may increase the risk that patients who are not infected may also receive broad-spectrum antibiotics, which will in turn facilitate the emergence of antimicrobial resistance [25]. According to the results of the current study, respondents believed that empiric antibiotic therapy should be restricted or stopped depending on the patient’s condition if no microorganisms are detected. Patients who may have sepsis or septic shock require a progressive treatment plan. The severity of the disease and a proper diagnosis should be considered together when determining which patients need rapid and aggressive therapy. When an infection is detected in a patient and there is a high probability of shock, immediate antibiotic therapy should be started. However, antibiotics should be discontinued immediately in situations when shock or fast exacerbation is not due to a bacterial infection [26].

In the present study, the majority of healthcare professionals were aware of the bundle elements to administer broad-spectrum antibiotics, need for blood culture before the antibiotic use, and blood lactate measurement. A similar study revealed that physicians considered bacterial culture to be the best technique for identifying sepsis [14]. In contrast to the present findings, hemodynamic monitoring was the second best technique for identifying sepsis in other studies [14,19]. Despite the fact that bacterial cultures are the most accurate way to identify bacterial illnesses, they impede early detection and treatment. Numerous studies have demonstrated that early identification and antibiotic therapy can lower sepsis-related mortality [3,20]. Therefore, for early detection of sepsis, various diagnostic modalities and strict monitoring of the early signs and symptoms should be implemented and used more frequently. A significant portion of respondents agreed that administration of antibiotics should be initiated at the earliest possible time, after the initial diagnosis of disease, which could be within 60 min. Consistently, the findings of a Turkish study showed that almost all the respondents agreed on the significance of the early administration of antibiotics [26]. Timely administration of fluids is vital for the treatment of patients [8]. Our study reported that most physicians were aware that fluid resuscitation methods must continue for the patient to recover. A study by Watkins et al. reported that the respondents decided to start their fluid resuscitation using crystalloids; the majority (95.6%) selected normal saline solution as their first preference, whereas 4.4% selected Ringer’s lactate solution [27].

Delay in diagnosis and treatment usually causes MOF, rapid progression to circulatory collapse, and ultimately death. Hence, a correct and well-timed identification will limit morbidity, decrease the cost of treatment, and improve patients’ outcomes [8]. In one Brazilian study, a lack of prompt diagnosis of the severity of sepsis by physicians was observed, highlighting the need for continuous professional activities directed towards the early acknowledgment and management of sepsis [28]. In the current study, the majority considered that delay in the identification of sepsis patients is the major barrier to starting timely treatment. It is reported that in high-risk sepsis patients, the bacterial cultures should be investigated immediately as soon as the patient arrives in the hospital and treatment should be started immediately with a broad-spectrum IV antibiotic along with administration of IV fluid that maintains blood sugar level and blood volume [29].

Based on the results of our study, we may conclude that respondents had an optimistic approach and frequently practice in accordance with the SSC guidelines. However, some respondents were not up-to-date with the most recent SSC guidelines in terms of knowledge and practices. There is a need for reinforcement on this issue to improve the awareness of sepsis treatment among healthcare professionals to combat sepsis and its effects. To lower mortality caused by incorrect diagnosis or poor management, frequent training programs in sepsis management could be introduced. The effectiveness of educational campaigns has been evaluated in several studies [14,18]. It has been shown that implementation of sepsis guidelines has a positive impact on sepsis management outcomes. Hence, patients could be allocated higher triage urgency codes and start to receive earlier antibiotic treatment. Significantly, the study findings highlight that efforts to support physicians to achieve a more coherent understanding and better agreement to the treatment guidelines could impact their clinical decision-making and patient outcomes.

Limitations of the study included the fact that data were obtained only from hospitals in Karachi, along with a cross-sectional study design, in addition to recall bias and selection bias. The current study has a lower response rate, which might be due to the demanding work schedules of physicians, which hinders physicians in responding to research and health surveys. Moreover, the lack of exploration in this area of the SSC bundle in the region makes it difficult to make a sweeping statement of the study findings at the national level. Further studies should be conducted on a national scale to authenticate and verify these results.

## 5. Conclusions

This study concludes that physicians considered sepsis an important part of the mortality burden in the healthcare system of Pakistan. Regardless of knowing the alarmingly high number of sepsis-related fatalities and impairments, some physicians had relatively little knowledge of the treatment strategies as suggested by the recommended guidelines. There is a need to actualize the gap between their insights and practices for combating sepsis and its consequences, as the severity of delays and lack of implementation of key diagnostic tests could derail the improvement efforts in the initial management of sepsis patients arriving in emergency rooms.

## Figures and Tables

**Table 1:** Demographic characteristics of the study population.

Characteristics	Frequency (*n*, %)
Gender	
Male	140 (56.9)
Female	106 (43.0)
Type of healthcare organization	
Private	168 (68.2)
Public sector	78 (31.7)
Place of work	
General hospital	177 (71.9)
Specialized hospital	69 (28.0)
Experience (years)	
Less than 5	118 (47.9)
6–10	66 (26.8)
11–15	36 (14.6)
16–20	20 (8.1)
Over 20	6 (2.4)
Position	
Consultant	53 (21.5)
General practitioner	106 (43.0)
Resident medical officer	87 (35.3)

**Table 2:** Physicians’ knowledge regarding the Surviving Sepsis Campaign Bundle elements.

Bundle Elements	Correct Responses (*n*, %)	Gender	Type of Healthcare Organization	Place of Work	Experience	Position
Definition of sepsis and septic shock	187 (76.0)					
Difference between sepsis and septic shock	175 (71.1)		0.002		0.005	
Threshold of blood lactate levels in sepsis	186 (75.6)					
Use of vasopressors if hypotensive during or after fluid resuscitation	91 (36.9)		0.001	0.01	0.034	<0.0001
Blood culture prior to administering antibiotics	216 (87.8)				0.002	
Administering broad-spectrum antibiotics	221 (89.8)		<0.0001		<0.0001	
Administration of 30 mL/kg of IV crystalloid fluid for hypoperfusion	89 (36.1)			0.006		
Target mean arterial blood pressure	142 (57.7)	<0.0001				
Target central venous pressure	154 (62.6)		0.006	0.004	<0.0001	
Target central venous oxygen saturation	29 (11.7)					0.008

Comparisons where the *p*-value was *p* ≥ 0.05 were not reported.

**Table 3:** Physicians’ attitude towards the Surviving Sepsis Campaign (SSC) Bundle.

To What Extent Do You Agree or Disagree with the following Statements Regarding Sepsis and Septic Shock?	Strongly Agree	Agree	Neutral	Disagree	Strongly Disagree	Mean ± SD
	*n*, %					
They are medical emergencies that needs immediate treatment and resuscitation.	50 (20.3)	154 (62.6)	30 (12.2)	10 (4.1)	2 (0.8)	3.98 ± 0.75
A performance improvement program for sepsis in hospital systems should include sepsis screening for critically ill, high-risk patients.	56 (22.8)	136 (55.3)	36 (14.6)	12 (4.9)	6 (2.4)	3.91 ± 0.89
Microbial cultures (including blood) should be obtained before starting antibiotic therapy.	68 (27.6)	122 (49.6)	50 (20.3)	6 (2.4)	0 (0)	4.02 ± 0.76
Administration of IV antibiotics should be initiated as soon as possible after recognition and ideally within one hour.	80 (32.5)	126 (51.2)	36 (14.6)	4 (1.6)	0 (0)	4.15 ± 0.72
Empiric broad-spectrum therapy with one or more antibiotics should be started to cover all likely pathogens.	60 (24.4)	126 (51.2)	44 (17.9)	14 (5.7)	2 (0.8)	4.11 ± 1.98
If no pathogens are found, empiric antibiotic therapy should be narrowed or discontinued based on the patient’s condition.	46 (18.7)	132 (53.7)	52 (21.1)	16 (6.5)	0 (0)	3.85 ± 0.80
Daily assessment (laboratory assessment) for de-escalation of antibiotic therapy in children with septic shock or sepsis-related organ failure should be considered.	50 (20.3)	100 (40.7)	60 (24.4)	32 (13)	4 (1.6)	3.65 ± 1.00
Duration of antibiotic therapy should be determined according to the site of infection, the microbiological etiology and the patient’s response to treatment.	46 (18.7)	132 (53.7)	42 (17.1)	24 (9.8)	2 (0.8)	3.80 ± 0.89
Dosing strategies of antibiotics should be optimized based on accepted pharmacokinetic/pharmacodynamic principles and specific drug properties.	82 (33.3)	124 (50.4)	28 (11.4)	12 (4.9)	0 (0)	4.12 ± 0.80
Balanced crystalloid solutions should be used for resuscitation rather than regular saline.	50 (20.3)	108 (43.9)	50 (20.3)	26 (10.6)	12 (4.9)	3.64 ± 1.07
Norepinephrine is considered as the first-choice vasopressor.	78 (31.7)	102 (41.5)	46 (18.7)	20 (8.1)	0 (0)	3.97 ± 0.91
Dopamine can be used as an alternate vasopressor to norepinephrine only in highly selected patients.	42 (17.1)	84 (34.1)	78 (31.7)	40 (16.3)	2 (0.8)	3.50 ± 0.99
IV corticosteroids should be used for people who are in septic shock and need vasopressor therapy on a regular basis.	30 (12.2)	120 (48.8)	66 (26.8)	26 (10.6)	4 (1.6)	3.59 ± 0.89
Instead of delaying to administer vasopressors until a central venous access is established, begin them peripherally to raise mean arterial pressure.	76 (30.9)	122 (49.6)	30 (12.2)	18 (7.3)	0 (0)	4.04 ± 0.85
Sepsis or septic shock survivors be evaluated and followed up on for physical, mental, and emotional issues after discharge from the hospital.	46 (18.7)	132 (53.7)	52 (21.1)	16 (6.5)	0 (0)	3.85 ± 0.80

## Data Availability

All data generated during the study are presented in this paper.
